# Building Capacity for Cyberbiosecurity Training

**DOI:** 10.3389/fbioe.2019.00112

**Published:** 2019-06-26

**Authors:** Lauren C. Richardson, Stephen M. Lewis, Ryan N. Burnette

**Affiliations:** Merrick and Company, Arlington, TX, United States

**Keywords:** cyberbiosecurity, biosecurity, cybersecurity, training, risk, threat, biosafety, capacity building (including competencies)

## Abstract

Cyberbiosecurity lies at the intersection of cybersecurity and biosecurity and addresses the protection of valuable biological material and associated information. As an emerging concept, cyberbiosecurity requires the integration of training strategies targeted to both current and future professionals; as well as an increased awareness in the wider stakeholder community. As the discrete discipline of cyberbiosecurity continues to develop, initial training efforts are likely to include workshops and specialized training that bridge the disciplines of information technology (IT) and life sciences. Potential threats, risks, and vulnerabilities will be defined, cooperative relationships formed, and collaborative solutions developed. As the scope of the training framework for assessing potential threats is adapted to various audiences, in-service trainings will ensure awareness and understanding of threats relevant to specific industries. This framework may also be incorporated into existing curricula across IT and science fields. The scope of potential threats is vast, and eventual specialization will likely fall within the realm of IT professionals, who carry the capability for action. In this paper, we identify stakeholders in the development of cyberbiosecurity training; discuss current training methods, educational requirements, and credentialing for professionals in cybersecurity, biosecurity, and life sciences; suggest mechanisms for integration of cyberbiosecurity training into existing training approaches; and discuss potential for future development of specialized professionals.

## Introduction

Cyberbiosecurity is a new, multidisciplinary concept with potentially significant impacts on the bioeconomy. Cyberbiosecurity addresses the potential for actual malicious destruction, misuse, or exploitation of valuable information, processes, and material at the interface of the life sciences and digital worlds, requiring an understanding of both (Richardson et al., 2019). Though the scope and definition of potential components continues to be refined and expanded, a common language and framework for the training and growth of a cadre of professionals is needed. Here, we propose a potential pathway for the development of cyberbiosecurity training.

## Identifying Cyberbiosecurity Stakeholders

This intersection of cybersecurity and biosecurity has the potential to affect organizations in multiple different fields, from agriculture and manufacturing to healthcare. Though many stakeholders possess a potential interest in the outcomes of cyberbiosecurity, a relatively small subset of individuals are well-suited to its execution. Due to the multidisciplinary nature of this field, those who conduct assessment, protection, and mitigation of cyberbiosecurity vulnerabilities should be well-versed in both the life sciences and information technology.

Today, biosecurity typically falls under the purview of institutional security and biosafety professionals, working together with external biosecurity assessors. It is generally understood that biosecurity requires an understanding of applicable assets (i.e., valuable biological materials and associated data) as well as an understanding of the threat landscape (e.g., negligent scientists without malicious intent, actors with targeted intent of theft or destruction for specific gain, and anarchic disruptors intent on disturbance of the system or organization). Cybersecurity is maintained at an institutional level by information technology professionals with myriad foundational knowledge bases in network security, systems engineering, on-site training in specific protection of systems within an organization.

Developing professionals in this nascent field requires training that draws—at least initially—from disparate disciplines within the life sciences and information technology. An individual with a thorough understanding of information technology and cybersecurity, plus a background in biological sciences, can be taught the basic tenets of risk, threat, and vulnerability assessment to achieve a comprehensive cyberbiosecurity base of knowledge.

## Status of Curricula in the Cybersecurity and Biorisk Management Fields

Training in the field of information technology is varied but well-established. While it is certainly possible to become an expert in one of the myriad IT fields via traditional education (i.e., university or vocational training programs), it is not required: many pathways and opportunities exist toward becoming a trained expert in one of the disciplines within the broader field of IT. Depending on the IT discipline in which one wishes to specialize, there are a number of training programs designed to teach individuals with little to no experience. In a traditional academic environment, individuals may choose to major in a computer engineering program, specializing in one of a range of disciplines, from network engineering to software development. Unlike the life sciences, however, IT expertise can also be gained through a less formal path: an individual may choose to attend a training program hosted by a non-academic organization (e.g., Computing Technology Industry Association). Individuals are also able to specialize in cybersecurity through academic and industry training programs. Further, many experts in the field of information technology obtain their primary education and experience through informal, hands-on training in one of a few domains, such as networking, cybersecurity, development, or systems engineering. With respect to cybersecurity training, many professionals also get their experience on the job; that being said, the International Information System Security Certification Consortium (ISC^2^) offers both training and a credentialing system aimed at standardizing topics of expertise, including security and risk management, asset security, security operations, security assessment and testing. These subjects are deeply congruent with the training and on-the-job experience offered within the fields of biorisk management and security.

In the life sciences, biosecurity training has historically been comprised of a varied and blended approach of teaching methodologies, including traditional classroom-based, non-traditional classroom-based (e.g., active learning, hands-on workshops), web-based/ online/ on-demand modules, train-the-trainer, on-the-job training, and others. Deciding which training methods to employ rests largely on considerations of (1) expected mastery of content and (2) proficiency of employing the information beyond the training. Further, the type of training content plays a significant role in dictating appropriate training approaches, frequency, and duration.

As science-based disciplines with a backbone in the biological sciences, biosafety and biosecurity benefit from serving a niche community of professionals and students. Like cybersecurity, the foundation of both resides in the broader discipline of risk management; biosafety is in fact a scientific-oriented field of risk assessment, mitigation, and management. Biosecurity, meanwhile, finds its roots in the field of threat assessment and management. Combined, the two disciplines converge at overall biorisk management (Burnette, 2013; Salerno and Gaudioso, 2015). Training in these areas has been largely developed by trade practitioners and official and unofficial repositories of training content; programs are maintained by professional and international organizations (e.g., ABSA International, International Federation of Biosafety Organizations). Effective organization of biosecurity training—as well as the training approaches themselves—remains in development (Minehata et al., 2013; Nixdorff, 2013).

## Convergence of Disparate Professional Fields and Curricula

A cyberbiosecurity professional is a practitioner with requisite foundational understanding of biological science principles and practice, fluency in IT lexicon and management, and concept mastery of risk and threat assessment. While this is an appropriate foundation for the cyberbiosecurity professional, additional understanding of a relevant field of practice (e.g., healthcare, pharmaceuticals manufacture) will be required for comprehensive understanding of field-specific vulnerabilities, as well as the ability to develop and promote mitigation strategies to address risks and threats to applicable assets. An ideal candidate may be an individual with a university degree in biology, or they may possess a combination of technical and professional training in IT, post-graduate training in biorisk management, and on-the-job training in cybersecurity. These unique and historically disconnected disciplines are rapidly converging in many sectors. Despite the recent emergence of cyberbiosecurity as a discrete discipline, many professionals possess overlapping skill sets.

## End-State of Successful Convergence

The professional development of specialists in this field will likely be similar to that of similar risk- and threat-based professions in the sciences, such as biosafety or industrial hygiene. Professionals in these fields typically receive training that includes university education in a basic science, post-secondary training in the field, and on-the-job training specific to their organization and position. Though large organizations may be well-served to employ specialized cyberbiosecurity professionals, it is likely most entities will have neither the resources nor the need for full-time employment, and the pool of available personnel will remain small.

Like similar fields, the specialist is not the only player with significant impact: biosafety, biosecurity, and cybersecurity professionals not specialized in cyberbiosecurity will potentially play a much larger role than the specialist, as they will be working on the front lines to recognize and address issues that threaten the science, data, and automation interface with the workforce and the public. Much like the executor of mitigations and corrective actions following a biosecurity audit, these professionals will likely bear the responsibility for following through on necessary measures to ensure sufficient cybersecurity within an organization or facility.

To achieve this end-state, scientific, IT, and security professionals must understand the requirements for and consequences of compliance with protocols and systems directed to address cyberbiosecurity. Just as scientists receive training in and comply with practices and policies to ensure biosafety, biosecurity, and cybersecurity as appropriate to their positions, they should also receive some degree of training in cyberbiosecurity.

## Standardization of Professional Training

Standardization of professional training—often resulting in credentialing—is well-established in the fields of biosafety and cybersecurity. ABSA International (formerly the American Biological Safety Association)—in conjunction with the American Society for Microbiology— has developed and maintained dual credentialing programs for biosafety professionals: the Registered Biosafety Professional (RBP) and the Certified Biological Safety Professional (CBSP) programs are experience and exam-based, respectively. Similarly, the International Federation of Biosafety Associations (IFBA) offers credentials in both biosafety and biosecurity. Both of these organizations offer a variety of training programs and curricula that provide foundations toward these credentials. Recently, ABSA International has undertaken an exploratory stance on the development of a biosecurity credentialing program somewhat analogous to the RBP and CBSP. However, with the exception of the IFBA certificate in biosecurity, no standardized biosecurity curricula or credential has been developed and implemented. This is in part due to the fact that biosafety has been a recognized scientific discipline for several decades, during which time biosecurity, as a discrete field of practice, remains inadequately defined.

## Development Process

### Need for Awareness and Definition

Today, there is limited awareness of concepts associated with cyberbiosecurity and their potential impacts on the bioeconomy. Significant change is required to move to an end state in which comprehensive management of cyberbiosecurity threats is integrated into existing organizations and systems. In the initial stages, this includes raising awareness regarding risks, threats, and vulnerabilities associated with cyberbiosecurity across many disparate sectors. Professionals within the risk and threat assessment communities are valuable allies and assets in identifying concepts, strategizing for integration, and sculpting the practice of cyberbiosecurity.

Important first steps have been taken in assessment of the potential impacts on the bioeconomy (Murch et al., 2018; Peccoud et al., 2018), definition of the threat landscape (Richardson et al., 2019), and assembly of professionals to begin to describe the field (Murch, 2017). Additional efforts are still needed: some will be described throughout this series, but more discourse is required to define the needs, impacts, and limitations of the field. Though cyberbiosecurity is not a fully-established field, integration of many of the concepts described can be easily integrated into existing training at the universities, technical trainings, and professional continuing education across related fields.

### Integration Into Existing Training

It stands to reason that the general field of information technology and cybersecurity is substantially larger that the field of biorisk management. This is especially true with regard to the number of extant professionals, curricula, and credentialing programs; as well as their overall applicability and integration into innumerable industries. In short, IT touches almost every aspect of daily existence. The same is not necessarily true for biorisk management, which remains a highly-specialized field of practice in discrete environments. From this, it can be inferred that incorporating elements of cybersecurity training into life sciences and biorisk management training would be a logical first step toward integrating seemingly disparate curricula. This argument is bolstered in the U.S. by the fact that the U.S. Federal Select Agent Program has stringent requirements surrounding appropriate access of information regarding biological select agents and toxins. This requires institutions with biological select agents and toxins to conform with a certain threshold of cybersecurity ^1^^,^^2^^,^^3^. An achievable goal in support of awareness and definition—as well as in support of building a developing repository of curricula—is to provide existing, relevant cybersecurity training to professionals in the biorisk management field.

Cybersecurity training is widely available via academic and industry-led curricula. There are also myriad massive online open courses (MOOC) available through credible services, some with linkages to universities. Additionally, many universities are offering formal education, including master's programs via remote, online programs; for which accessibility and affordability are key components. As the demand for cybersecurity experts at the intersection of IT and life sciences continues to grow, it is not difficult to imagine that cyberbiosecurity courses will become popular offerings at online and traditional universities.

## Curriculum Development

A significant challenge in the burgeoning field of cyberbiosecurity is the development of a curriculum relevant from both discipline and market perspectives. It is reasonable to assume that curricula will be driven by both technical needs (e.g., discrete and relevant content representative of the needs of practitioners) and by the market pool of would-be professionals and students supporting the industry. Given the breadth of existing curriculum in the fields of biosafety, biosecurity, and cybersecurity, it stands to reason that a comprehensive requirements identification process can be conducted to cross-reference the three disparate disciplines at technical and content levels. Further, this requirements development process is likely to reveal substantial information about the market itself. It is anticipated that many independent requirements already in existence (such as biosafety and cybersecurity curricula), will reveal common, logically-linked themes. However, new requirements not currently captured in any singular discipline are likely to be identified; new content will have to be developed to constitute a body of knowledge representative of the field as it is developing today, with a focus on future development.

Like many developing fields, the establishment of instructors, trainers, and teaching professionals capable of maintaining a curriculum focused on industry needs is likely to be one of the more challenging aspects of training in the cyberbiosecurity field; the general lack of professionals who are equally expert in the biological sciences and cybersecurity practices speaks to this challenge. This is also demonstrated by the fact that biosecurity has yet to be adequately codified in the fields it touches (such as laboratories, agriculture, and personalized medicine, among others). The result is a general lack of professionals who can justify their status as a “biosecurity professional.” Often, credentials help their respective fields maintain their relevance. For example, we see Registered Biological Safety Professional (RBP) and/or Certified Biological Safety Professional (CBSP) listed as a requirement within job descriptions for biosafety personnel. Accordingly, it is often an expectation that qualified teaching staff have the same credentials. While analogous professional biosecurity credentials will be considered, it is premature to assume this credential will offer any specialization toward cybersecurity.

## Credentialing

Credentialing frameworks may need to be designed and implemented in order to identify and educate interested practitioners working within the emerging field of cyberbiosecurity. There is currently a high barrier to learning concepts in each of the cybersecurity and biosecurity disciplines as independent entities; interested parties willing to take the steps toward an applied career in cyberbiosecurity will need to understand the unique challenges that exist within both disciplines. The implementation of credentialing systems may be beneficial toward standardization of the knowledge base required to be an expert in the field. There are also sub-fields of each discipline ostensibly more relevant to the emergence of cyberbiosecurity (e.g., bioinformatics, network security, sequence origin identification, cloud laboratories, machine learning). These sub-fields could be used to develop a credentialing framework (distinct from existing frameworks today) via employing each component as separate training module. Alternatively, it may be prudent for leaders in this emerging field to partner with existing and well-established organizations in cybersecurity credentialing (e.g., International Information System Security Certification Consortium) to develop and implement a cyberbiosecurity training program. At the time of this writing, there is no credentialing system established for biosecurity: it remains in development by organizations like the American Biological Safety Association. Discrete training courses and workshops could also be implemented as a starting point to introduce the need for a credential, as well as to receive support from cybersecurity and biosecurity experts.

## The Path Forward

A new discipline is not built in a matter of months: it grows organically from existing, related fields, and is supported by advocates and experts who recognize its significance and distinction. Additional workshops, papers, and open fora will encourage collaboration for further definition of relevant concepts. Introduction of these concepts should be presented at various professional symposia and conferences in order to raise awareness, introduce ideas for integration, and bring together interested individuals. From these interested parties, a working group may consolidate in order to develop educational materials that can be integrated into professional and academic organizations.

A working group with experts from multiple fields will define gaps and areas for integration across sectors and personnel within organizations—potentially leading to the development of a formalized cyberbiosecurity curriculum. A well-defined curriculum may be easily integrated into an academic or technical training system, as appropriate. At this stage, a credentialing mechanism is likely to emerge; however, it is challenging to predict whether this curriculum and credentialing system will fall within the scope of the life sciences or IT. Foundational elements of a cyberbiosecurity credentialing framework are currently in development by thought leaders spanning both IT and life sciences disciplines. The biosecurity credential, currently under evaluation by ABSA International, accounts for cybersecurity elements as a basis for and component of an industry credentialing program. Specifications for a credentialing framework will be further developed and include core competencies, such as physical security, regulations and compliance, biorisk management, secure network architecture, identity management, disaster recovery, and security operations in life sciences facilities.

Concepts addressed in cyberbiosecurity span myriad disciplines, so an open dialogue between subject matter experts, as well as affected stakeholders, is required. Professionals in cyberbiosecurity will require not only expertise and training in science and technology concepts, but also the ability to effectively execute a new form of technical communication across disciplines and organizations to achieve comprehensive solutions.

## Author Contributions

LR, SL, and RB contributed conception and wrote sections of the manuscript. All authors contributed to manuscript revision, read, and approved the submitted version.

### Conflict of Interest Statement

LR, SL, and RB were employed by Merrick and Company.

## Footnotes

^1^(2005). *Possession, Use, and Transfer of Select Agents and Toxins*, in *7 § 331*. United States Code of Federal Regulations.

^2^(2005). *Possession, Use, and Transfer of Select Agents and Toxins, in 9 § 121., United States Code of Federal* Regulations.

^3^(2005). *Select Agents and Toxins, in 42 § 73., United States Code of Federal* Regulations.
